# Supplementary material for: Titanium Surfaces with a Laser-Produced Microchannel Structure Enhance Pre-Osteoblast Proliferation, Maturation, and Extracellular Mineralization In Vitro
Source: Int J Mol Sci. 2024 Mar 16;25(6):3388. doi: 10.3390/ijms25063388 (PMC10970313; doi:10.3390/ijms25063388)
Supplement: Supplementary file 1 [file ijms-25-03388-s001.zip › ijms-2892886-supplementary.pdf]

Supplemental Methods

Energy-dispersive X-ray spectroscopy assay

The G-, SLA-, and L-titanium plates were soaked in simulated body fluid for 4 or 18 days. After the plates were air-dried, the atomic percentage of calcium and phosphorus sediment was evaluated by performing an energy-dispersive X-ray spectroscopy (EDS) assay.

Supplemental Results

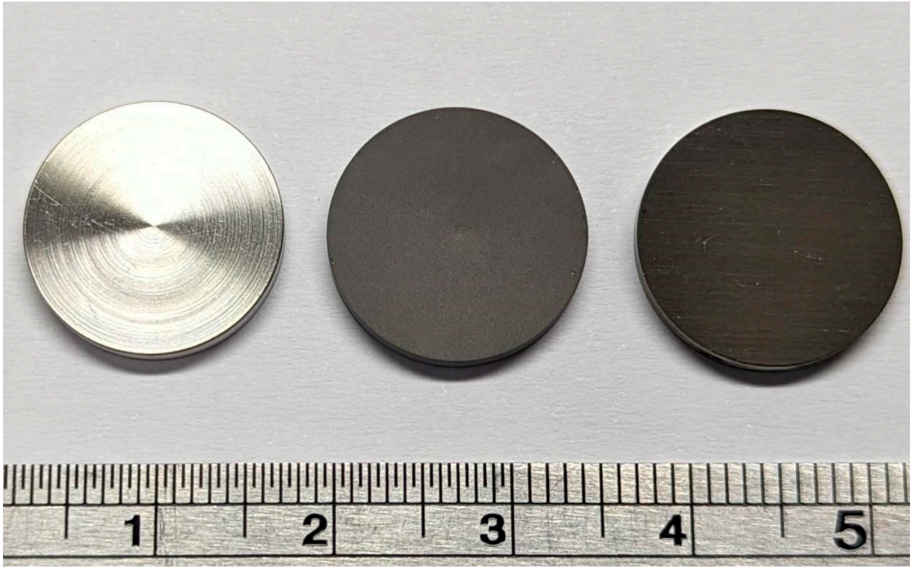

Figure S1. The titanium plates with G-, SLA-, and L-treated surfaces The titanium plates were treated with G, SLA, and L from left to right. The interval between numbers is 1 centimeter.

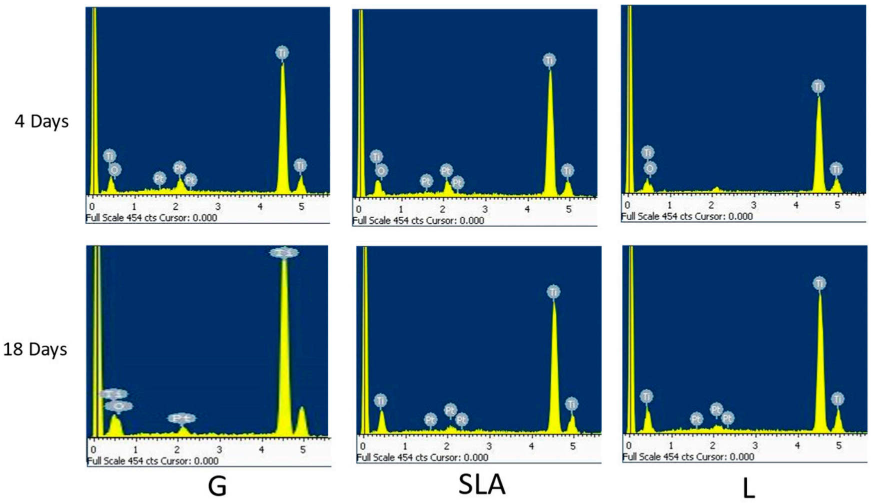

Figure S2. Calcium and phosphorus sediment was similar for G-, SLA-, and L-treated titanium surfaces. The titanium plates with G-, SLA-, and L-treated surfaces were soaked in simulated body fluid for 4 or 18 days. After air-drying, the experimental plates were subjected to an energy-dispersive X-ray spectroscopy (EDS) assay.
